# Serological evaluation for Chagas disease in migrants from Latin American countries resident in Rome, Italy

**DOI:** 10.1186/s12879-018-3118-5

**Published:** 2018-05-08

**Authors:** Stefania Pane, Maria Letizia Giancola, Pierluca Piselli, Angela Corpolongo, Ernestina Repetto, Rita Bellagamba, Claudia Cimaglia, Stefania Carrara, Piero Ghirga, Alessandra Oliva, Nazario Bevilacqua, Ahmad Al Rousan, Carla Nisii, Giuseppe Ippolito, Emanuele Nicastri

**Affiliations:** 1 National Institute for Infectious Diseases “Lazzaro Spallanzani”, IRCCS, Via Portuense, 292, 00149 Rome, Italy; 2 Operational Center Bruxelles, Mèdecins Sans Frontières, Bruxelles, Belgium

**Keywords:** Chagas disease, Trypanosoma cruzi, Seroprevalence, Migrants, Italy

## Abstract

**Background:**

Chagas disease (CD) is a systemic parasitic infection caused by the protozoan *Trypanosoma cruzi,* whose chronic phase may lead to cardiac and intestinal disorders. Endemic in Latin America where it is transmitted mainly by vectors, large-scale migrations to other countries have turned CD into a global health problem because of its alternative transmission routes through blood transfusion, tissue transplantation, or congenital. Aim of this study was to compare the performance of two commercially available tests for serological diagnosis of CD in a group of Latin American migrants living in a non-endemic setting (Rome, Italy). The study was based on a cross-sectional analysis of seroprevalence in this group. Epidemiological risk factors associated to CD were also evaluated in this study population.

**Methods:**

The present study was conducted on 368 subjects from the Latin American community resident in Rome. Following WHO guidelines, we employed a diagnostic strategy based on two tests to detect IgG antibodies against *T. cruzi* in the blood (a lysate antigen-based ELISA and a chemiluminescent microparticle CMIA composed of multiple recombinant antigens), followed by a third test (an immunochromatographic assay) on discordant samples.

**Results:**

Our diagnostic approach produced 319/368 (86.7%) concordant negative and 30/368 (8.1%) concordant positive results after the first screening. Discrepancies were obtained for 19/368 (5.2%) samples that were tested using the third assay, obtaining 2 more positive and 17 negative results. The final count of positive samples was 32/368 (8.7% of the tested population). Increasing age, birth in Bolivia, and previous residence in a mud house were independent factors associated with *T. cruzi* positive serology.

**Conclusions:**

Serological diagnosis of CD is still challenging, because of the lack of a reference standard serological assay for diagnosis. Our results reaffirm the importance of performing CD screening in non-endemic countries; employing a fully automated and highly sensitive CMIA assay first could be a cost- and resource-effective strategy for mass screening of low-risk patients. However, our results also suggest that the WHO strategy of using two different serological assays, combined with epidemiological information, remains the best approach for patients coming from endemic countries.

## Background

Chagas disease (CD), also known as American Trypanosomiasis, is caused by the protozoan *Trypanosoma cruzi* (*T. cruzi*), usually transmitted by infected triatomine bugs [1, 2]. The disease is endemic in Latin America where humans become infected through contact with the faeces of the blood-sucking vectors, which contain the infective stages of the parasite. While the acute illness is frequently asymptomatic, the chronic phase that follows is characterized, in about 10–30% of cases, by disorders mainly involving the heart and the gastrointestinal tract [1, 2], and it is estimated by the World Health Organization (WHO) that about 8 million people are infected worldwide and are at risk of developing such complications [3]. Although historically confined to the Americas, especially rural areas of Latin America where poor housing conditions have promoted contact with infected vectors, migrations from endemic countries have led in recent years to the appearance of the disease also in other parts of the world [4–7]. In both endemic and non-endemic countries, transmission of *T. cruzi* may also occur through blood transfusion, tissue transplantation, or congenitally from mother to infant [1, 2]; these alternative infection routes are likely to occur in non-endemic settings and have turned CD into a global health problem [5, 8].

While vector control programmes and improved housing conditions have led to a reduction in the incidence of CD in Latin America, diagnosing chronic and often asymptomatic patients has for a long time been, and still is, a major challenge. Because of low and intermittent parasitaemia, diagnosing the disease in its chronic stage relies on serological methods that detect antibodies directed against *T. cruzi* [1, 2, 9]. Such methods are classically divided in two categories: conventional (based on antigens obtained from the whole parasite), and non-conventional (based on the use of recombinant antigens). Because of the lack of a single reference standard test, the possibility of cross-reactivity, and the biological diversity and genetic polymorphism of *T. cruzi*, which is due to the existence of six discrete typing units (DTUs) of the parasite in different geographical areas [10]*,* the WHO recommends that a diagnosis of chronic CD be based on two positive results obtained using two different methods: a conventional test followed by a non-conventional assay [9]. Serological diagnosis remains a challenge and screening schemes have been implemented only recently, mainly for blood and organ donors, pregnant women, and newborns [11–13], in an effort to control the transmission of CD [14].

This study was based on a cross-sectional analysis of seroprevalence in a group of Latin American migrants living in Rome, Italy, and its aim was to compare the performance of two commercially available serology tests (ELISA and CMIA) for diagnosing CD in a non-endemic setting. A third assay (ICT) was used in case of discordant results, according to WHO recommendations. Epidemiological risk factors for *T. cruzi* infection were also evaluated in this population sample, to verify the validity of the diagnostic strategy employed.

## Methods

Between February and June 2014, the ‘Lazzaro Spallanzani’ National Institute for Infectious Diseases, in collaboration with the Italian Mission of the international non-governmental organization *Médecins sans frontières (MSF)*, promoted a screening campaign to detect anti-*T. cruzi* IgG antibodies among migrants from endemic Latin American countries residing in the rural and urban areas of Rome, Italy. *MSF* contacted and networked with Latin American embassies, consular authorities and cultural associations to plan and implement the community approach to raise awareness on health promotion issues. The activity was conducted by specifically trained professionals with the aid of visual materials for reaching people and providing information on the screening. The potential target of the screening campaign was the population coming from Latin American countries, which according to an official estimate amounted to 37,197 persons in 2014 [15]. All individuals contacted were aware that their participation would be voluntary, open and free. No selection of participants was made, and the only inclusion criterion was their Latin American origin.

A socio-demographic questionnaire designed to identify factors associated with *T. cruzi* infection was administered to each participant by an infectious diseases specialist, and the results were anonymized and recorded in an electronic database. The information collected included clinical and epidemiological characteristics such as age, sex, country of origin and residence in the country of origin, as well as the date of arrival in Italy and any history of Chagas disease or whether migrants had received blood transfusions, in endemic countries or not.

CD diagnosis was based on two concordant positive serological tests. As per WHO guidelines [9], the chosen methods were based on different principles and antigens: i) the lysate antigen-based ELISA (BioELISA Chagas III, BiosChile, Santiago, Chile) is a solid phase immunoassay that uses *T. cruzi* extracts containing highly immunogenic membrane antigens attached to the microtiter plate; ii) the chemiluminescent microparticle immunoassay (CMIA, Architect Chagas®, Abbott Diagnostics) is a fully automated assay based on recombinant proteins FP3, FP6, FP10, and TcF, which represent 14 distinct antigenic regions; iii) an immunochromatographic (ICT) assay based on multi-epitope recombinant antigens**,** comprising a total of nine different epitopes, was used in case of discordant result (Chagas Quick Test ICT, Cypress Diagnostics, Langdorp, Belgium). These tests were commercially available in Europe already at the time of the screening and their respective manufacturers declared a 100% sensitivity and specificity for the ELISA and ICT assays, 99% for the CMIA method. A blood sample was drawn from enrolled individuals, and all assays were performed according to manufacturers’ instructions. Samples with index values > 1.1 (ELISA) and > 1 (CMIA) were considered positive, and the cut-off regions were as follows: 0.9–1.1 for the ELISA method, and 0.8–1.0 for the CMIA assay.

A clinical diagnostic and therapeutic protocol that included chest-X-ray, electrocardiogram, echocardiography, gastric and colon endoscopy and antiparasitic benznidazole therapy was offered to all patients that tested positive for CD.

### Statistical analysis

A descriptive analysis was conducted to better characterize the subjects enrolled in the study. Median values and interquartile ranges (IQR) were used to describe numerical variables, while counts and percentages were employed for qualitative variables. Chi-square (χ^2^) test (or Fisher’s exact test when applicable) or Mann–Whitney non-parametric tests were used to compare groups for categorical or continuous variables, respectively. As a measure of association, we calculated odds ratio (OR) and multivariable logistic regression odds ratio (MLR-OR) and their 95% confidence intervals (95% CI). The MLR was adjusted for variables significantly associated with Chagas infection in the univariate analysis (*p* < 0.05) forcing gender in the final model. All statistical analyses were performed using IBM SPSS Statistics version 24 (IBM Corp., Armonk, N.Y., USA).

### Ethical issues

The study protocol had been approved by the Ethics Committee of the Spallanzani Institute in October 2013 and written informed consent was obtained from all individuals at the time of screening.

## Results

During the 5-month screening programme, 368 patients were enrolled in the study. Of these, 264 (71.7%) were women (median age 43 years; IQR: 34–51 years) and 104 (28.3%) were men (median age 39 years; IQR: 31–47 years). Overall, the median age of the study population was 42 years (IQR: 33–51 years). The geographical distribution of included individuals was as follows: 115 subjects (31.2%) were from Bolivia, 123 (33.4%) from Ecuador, 62 (16.8%) from Peru, 28 (7.6%) from Colombia, 20 (5.4%) from other Latin American countries. Twenty additional individuals (5.4%) were born in Italy but were included because they were second-generation migrants or Italian nationals with a history of long-term residence in Latin American countries.

**Fig. 1:**
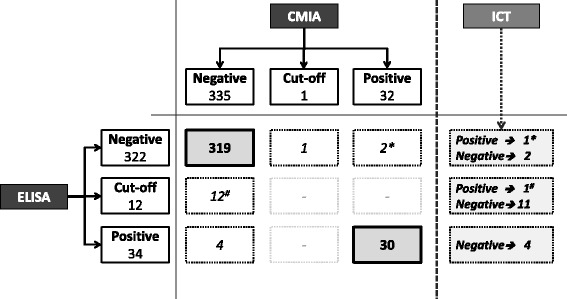
Results of *T. cruzi* antibodies according to the diagnostic strategy used. Results of the two serological tests (ELISA and CMIA) used for detecting *T. cruzi* antibodies in 368 individuals, defined as negative, cut-off (equivocal) and positive for both methods (left and top). The combined results are shown in the centre of the figure, where dotted boxes indicate discordant samples on which the third confirmatory assay (ICT) was carried out (right)**.** * indicates a discordant sample that was confirmed as positive by ICT**.** #indicates a sample that was negative by CMIA, equivocal by ELISA, but positive by ICT

Of the 368 individuals, 242 (65.8%) had lived in rural areas and 263 (71.5%) in mud houses; 23 (6.3%) reported a previous blood transfusion in an endemic country, and 15 (4.1%) provided at least one blood donation during their stay in Italy. All enrolled individuals were asymptomatic, except one who presented arrhythmias. Figure 1 reports the results according to the diagnostic strategy used, consisting of two different screening methods (ELISA and CMIA) and a third assay employed on discordant samples, in accordance with WHO guidelines [9]. According to the ELISA assay, 322 (87.5%) serum samples were found to be negative, 34 (9.2%) serum samples resulted positive and 12 (3.3%) were equivocal. Using the CMIA method, 335 (91.0%) samples were negative, 32 (8.7%) were positive and only one (0.3%) was equivocal. Concordant negative results for both ELISA and CMIA assays were obtained for 319 (86.7%) samples, while 30 (8.1%) were positive in both tests and were therefore considered unambiguously positive.

**Fig. 2:**
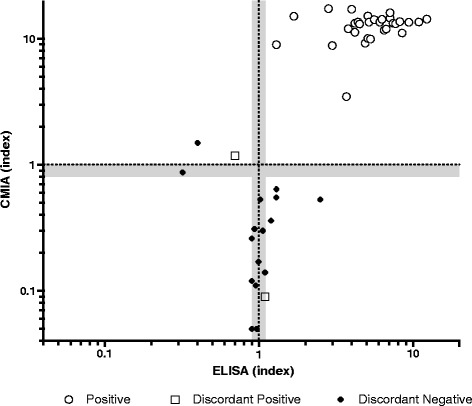
Dispersion graph of index values obtained for the ELISA and CMIA methods. Dispersion of results of the CMIA and ELISA assays, showing only concordant positive samples (white dots), discordant results that were confirmed as positive (white squares), or negative (black dots). With a gray area, both for CMIA and ELISA assays, ‘cut-off’ regions are highlighted: sample/cut-off ratio 0.8–1.0 for CMIA and 0.9–1.1 for the ELISA method

Overall, discordant results in the two screening tests were recorded for 19 samples (5.2%), which were investigated by the ICT assay. Positive results were obtained for 2/19 (10.5%) while the remaining 17 (89.5%) resulted negative. These two positive samples detected by ICT were added to the 30 positive results obtained by ELISA and CMIA, providing a cumulative number of 32 samples positive for *T. cruzi* antibodies, allowing to calculate an 8.7% overall anti-*T. cruzi* antibody positive rate in the study population. The dispersion graph in Fig. 2 shows the index values (defined as the ratio of the reading of the sample and the cut-off control) obtained for the ELISA and CMIA methods. As per manufacturers’ instructions, samples with index values > 1 (CMIA) and > 1.1 (ELISA) were considered positive. The cut-off regions are shown as grey areas, and were as follows: 0.9–1.1 for the ELISA method, and 0.8–1.0 for the CMIA assay.

Of the 32 *T. cruzi*-positive migrants, 27 subjects were born in Bolivia, providing a 23.5% *T. cruzi* positive serology rate among Bolivian migrants, 2 (6.2%) were born in El Salvador and 3 (3.1%) came from the following countries: Brazil, Colombia and Ecuador. Overall, subjects with a positive serological test for *T. cruzi* were more frequently of older age (*p* = 0.015), born in Bolivia (*p* < 0.001) and with a history of living in mud houses (*p* = 0.001) compared to individuals with negative results (Table 1).

**Table 1:** Characteristics of the 368 patients enrolled

			*T. cruzi* positive serology		*p* value
			Yes: N (%)	No: N (%)	
Total number		**368**	**32 (8.7)**	**336 (91.3)**	
Age, median (IQR) years		42 (33–51)	47 (40–52)	42 (32–50)	**0.015**
Years of residence in Italy, median (IQR) years		11 (7–14)	10 (7–13)	12 (8–14)	0.253
Gender	M	104	6 (5.8)	98 (94.2)	0.211
	F	264	26 (9.8)	238 (90.2)	
Subjects born in Bolivia	No	253	5 (2.0)	248 (98.0)	**< 0.001**
	Yes	115	27 (23.5)	88 (76.5)	
Residence in rural environment	No	126	8 (6.3)	118 (93.7)	0.249
	Yes	242	24 (9.9)	218 (90.1)	
Residence in mud houses	No	105	1 (1.0)	104 (99.0)	**0.001**
	Yes	263	31 (11.8)	232 (88.2)	
Previous blood transfusion in endemic countries	No	345	32 (9.3)	313 (90.7)	0.243
	Yes	23	0 (−)	23 (100.0)	
Blood donation in Italy	No	353	31 (8.8)	322 (91.2)	1.000
	Yes	15	1 (6.7)	14 (93.3)	

*IQR* Interquartile range; *p*-values < 0.05 are reported in bold face

At multivariable analysis, age per 10-years increase (OR = 1.9, *p* = 0.002), birth in Bolivia (OR = 22.1, p < 0.001), and previous residence in a mud house (OR = 12.1, *p* = 0.017), were all independent factors associated with *T. cruzi* infection (Table 2).

**Table 2:** Predictive factors of *Trypanosoma Cruzi* positive serology at multivariable analysis

		*T. cruzi* positive serology/Tot.	Multivariable analysis	
			MLR-OR (95% CI)	p-value
		**32/368**		
Age, by 10 years increase			1.98 (1.25–2.86)	**0.002**
Gender	M	6/104	1	0.375
	F	26/264	1.59 (0.57–4.42)	
Subjects born in Bolivia	No	5/253	1	**< 0.001**
	Yes	27/115	22.09 (7.34–66.44)	
Residence in mud houses	No	1/105	1	**0.017**
	Yes	31/263	12.13 (1.57–93.77)	

*MLR-OR* Multivariable logistic regression Odds Ratio; p-values < 0.05 are reported in bold face

## Discussion

CD is a public health concern because of the array of disorders it causes in the chronically infected; pregnant women and immunosuppressed individuals are especially vulnerable because of the high risk of infection for the unborn child [1, 2, 16], and the possibility of reactivation, respectively. The epidemiology of the disease has evolved dramatically in recent years because of increasing migrations of people from endemic to non-endemic areas, where the infection has become a public health concern [17]. Early diagnosis of *T. cruzi* infection in non-endemic countries is therefore of primary importance in order to control the transmission of the disease (whether by blood transfusion, organ transplant, or congenital route), and reduce reactivation in immunosuppressed individuals [1, 2, 17].

According to recent estimates, 100,000 people living in Europe could be infected by *T. cruzi* [5], and Italy would be one of the countries with the highest burden, hosting between 6000 and 12,000 infected individuals [18]. Although diagnosing CD in asymptomatic patients is a remarkable medical challenge especially in non-endemic countries, delaying or avoiding the onset of the cardiac and gastrointestinal complications that characterize chronic infection would have a great impact on the quality of life of the affected patients, and on public health systems.

A recent cost-effectiveness study reports that screening for *T. cruzi* in asymptomatic adults at risk of being infected, treating and following up those testing positive, is a cost-effective strategy in European countries with the highest number of immigrants from Latin America [19]. According to this study, the cost per quality-adjusted life-years (QALYs) gained is much lower than the currently accepted threshold of €30,000 per QALY in Spain and other European countries. Furthermore, the cost of adopting a *T. cruzi* screening is no higher than that reported for other common infectious diseases in migrants in Europe such as hepatitis B or C [20].

A suggestive epidemiology (having lived in a rural area of an endemic country, in adobe houses) is a known risk factor for CD and it is noteworthy that of the subjects with a positive *T. cruzi* serology in our study, all but one reported a history of living in a mud house and were therefore likely to have been exposed to triatomine bites. A high proportion of migrants originating from Andean countries are living in Rome [15] and our results among this selected subset of migrants confirm a higher proportion of positive tests for *T. cruzi*, as expected. Although the population studied was not representative of the whole community of Latin American migrants living in Rome, we observed an overall 8.7% prevalence, with a peak of 23.5% among Bolivian nationals. Although affected by a selection bias, these results are in agreement with seroprevalence data observed in previous works [5, 18, 21–23]. It must be also noted that our study was conducted on subjects enrolled on a voluntary basis, and it is likely that prior knowledge of CD or its presence in their family or country of origin may have played a role in their decision.

We chose to apply the ELISA and CMIA tests first, for reasons of rapidity and the possibility of automation. The ICT that was used on discordant samples was chosen because of its optimal sensitivity and specificity, as shown by a surveillance study conducted in Italy on Latin American blood donors [24]. We recorded 32 samples that were positive by two distinct serological methods and can be considered true positive cases of CD infection according to WHO guidance [9]. The double testing recommended by WHO could prove resource consuming when large numbers of samples need to be processed, as would be the case for endemic areas or blood banks where, in situations of different prevalence, predictive values could be markedly affected. Considering the lack of a gold standard for CD diagnosis and of a confirmatory test, especially in Europe, new-generation assays based on mixtures of recombinant antigens and highly sensitive detection techniques, such as chemiluminescence, have been developed recently. These assays, in addition to improving sensitivity and specificity compared to conventional methods based on whole parasite antigens, also offer the advantage of automation and rapidity. The CMIA method we employed has already been proposed as a possible single screening method based on results obtained on samples that had been pre-characterized using conventional techniques [12]. Our study on the other hand was conducted on asymptomatic subjects whose serological status was unknown and whose only reason for screening was their Latin American origin; our results confirm the validity of the assay, as 31 out of 32 (96.9%) positive individuals could be identified using the CMIA method alone, with a specificity of 99.7% (Fig. 1). For the ELISA test, results were slightly lower (sensitivity 93.8% and specificity 98.8%). The dispersion graph in Fig. 2 highlights more clearly how the ELISA assay gave more equivocal or false positive results (discordant negative) than the CMIA, and that all the concordant positive samples showed higher index values, representing a separate cluster in the upper right quadrant. The only false negative sample (none were recorded in the study by Abras et al.), which was equivocal in the ELISA assay (though with a 1.1 index value which represents the upper value of the ‘cut-off’ region) and confirmed positive by the ICT test, would not have been missed using a combined laboratory and epidemiological approach, as it belonged to a 54-year old woman who reported having lived in a mud house in Ecuador.

Diagnosis of chronic Chagas is challenging. Although a wide range of serological tests for clinical diagnosis of CD and for blood screening for *T. cruzi* are available today, the ideal single serological test, with optimal sensitivity and specificity, is not yet available. In clinical practice, most cases are serologically diagnosed using a combination of tests with high specificity (such as the IHA, or those employing recombinant antigens) and tests with high sensitivity (such as IFA or ELISA) [9].

In the most difficult cases, when the recombinant assays are not helpful in clarifying inconclusive or discordant cases, WHO suggests the use of western blot with trypomastigote excretory–secretory antigen (TESA) [9]. However, even the TESA assay showed some limitations and clarified only half of discordant cases in Europe [25].

## Conclusions

Our data confirm the importance of performing CD screening on all patients from Latin America. At present, serological diagnosis of CD remains a challenge. Given the lack of a reference gold standard, there is a need of quality control schemes, standardization of the diagnostic methods available, and of confirmatory assays for specific target population, especially in non-endemic countries. Although a validation on larger numbers of subjects is required, our results also suggest that in a non-endemic country individuals at high risk of *T. cruzi* infection, identified by the presence of at least two out of three risk factors identified in our analysis (older age, previous residence in a mud house, birth in Bolivia) should be processed according to the standard WHO recommendation. Low-risk subjects on the other hand could be screened by a sequential testing policy based on a first screening made by CMIA, in a cost and time sparing strategy. This study also underlines the need for European clinicians to consider CD screening for all asymptomatic Latin American nationals, especially in presence of determinant factors associated with *T. cruzi* infection [18, 21–23]. Finally, it would be helpful if European and national health agencies recognized and declared CD a rare and orphan disease, in order for it to gain more attention and specific plans to promote the knowledge, early diagnosis and sustainable access to treatment and care.

## Abbreviations

CD: Chagas Disease
CMIA: Chemiluminescent Microparticle Immuno Assay
ELISA: Enzyme-Linked Immunosorbent Assay
ICT: Immunochromatographic Test
IQR: Interquartile Range
*MSF*: *Médecins sans frontières*
*T. cruzi*: *Trypanosoma cruzi*
WHO: World Health Organization
